# Chemistry

**Published:** 1836-01

**Authors:** 


### CHEMISTRY.

### *Comparative Analysis of White and Grey Cerebral Matter.*

M. John is the only chemist who, in his analysis of the brain, has hitherto separately examined the grey and white matter. He has stated that the white matter contained more fat than the grey, and that its albumen was more firm. The following comparative analysis was made of the brain of one of the insane patients who died at the Salpêtrière.

*Entire Brain,* (Density = 1048.)

| | |
|---|---|
| Water . . . . | 77. |
| Albumen . . . | 9.6 |
| White fatty matter . . | 7.2 |
| Red fatty matter . . | 3.1 |
| Osmazome, lactic acid, and salts | 2.0 |
| Earthy phosphates . . | 1.1 |

| | *White Substance.* | | *Grey Substance.* |
|---|---|---|---|
| Water . . | 73.0 | .... | 85.0 |
| Albumen . . | 9.9 | .... | 7.5 |
| White fatty matter | 13.9 | .... | 1.0 |
| Red fatty matter . | 0.9 | .... | 3.7 |
| Osmazome, &c. . | 1.0 | .... | 1.4 |
| Earthy phosphates . | 1.3 | .... | 1.2 |

—*Journal de Chimie Médicale,* Août, 1835.

### *Solidification of Carbonic Acid Gas.*

At a meeting of the Academy of Sciences, October 12th, M. Thiloriet announced that he had reduced carbonic acid gas to a solid form. M. Arago, who read the letter from which the following particulars are extracted, stated that a commission had satisfied themselves of the correctness of the experiments.

Carbonic acid, which is gaseous at ordinary temperature and pressure, and liquid at 0° (C.) or 32° (F.), under a pressure of thirty-six atmospheres, becomes solid at a temperature of 100° (C.) below the freezing point, or (— 148° F.), and remains some minutes in this new condition, even when exposed to the air, without compression.

Its elasticity, which in a liquid state is so great that it produces an explosion equal to the same weight of gunpowder, is destroyed by solidification, and the new solid is gradually dissipated by evaporation. Another curious fact is, that this gas is solidified by its suddenly passing from a liquid to a gaseous state; the near approach of its molecules, which constitutes it a solid, being caused by the expansion of a liquid which occupies instantaneously a space four hundred times greater than its primitive volume. If a jet of carbonic acid is directed into a small glass vial, it becomes rapidly and almost entirely filled with a white flocculent powdery substance, which so strongly adheres to the glass that it cannot be removed unless the bottle is broken. A fragment of solid carbonic acid, slightly touched with the finger, glides rapidly over a polished surface, as if it were raised by the gaseous atmosphere which constantly surrounds it, until it entirely disappears. If a small quantity *(quelques décigrammes)* of this substance is introduced into a small flask, hermetically stopped, the interior becomes filled with a thick vapour, and the stopper is soon driven out violently. Solid carbonic acid is completely evaporated, and only rarely a slight humidity remains, which must be attributed to the action of the air on a very cold body, of which the temperature is below that at which mercury freezes. Its abundance, and the promptitude with which it is produced in cavities where neither air nor watery vapour held in it can penetrate, are characters that cannot be mistaken.—*Gazette Médicale de Paris,* No. 43; 24 Oct. 1835.

